# Representation in the American Medical Association

**Published:** 1851-07

**Authors:** Thos. H. Yardley

**Affiliations:** Philadelphia


### *Representation in the American Medical Association.* By T. H. Yardley, M. D., Philadelphia.

To the Editors of the Medical Examiner:

Gentlemen—I will be obliged if you will correct an error in your last number, in relation to the resolution offered by me at the late meeting of the "American Medical Association," in Charleston.

The following are the preamble and resolution as offered, and adopted by the Philadelphia County Medical Society, viz:

"Whereas, The Constitution of the American Medical Association, by providing for the reception of delegates from 'permanently organized Medical Societies, Medical Colleges, Hospitals, Lunatic Asylums, and other permanently organized Medical Institutions,' unjustly favours the profession in cities, where such institutions exist and are readily formed, and diminishes the importance and thereby discourages the establishment of County Medical Societies in rural districts;

*Resolved*, That the Constitution of said Association should be so amended as to admit only delegates from County or State Medical Societies."

While on the subject, I will, with your permission, correct an error which exists in regard to the object of those who introduced this resolution; we do not desire, as has been asserted, to exclude *Professors* from the Association; we wish, in the language of Dr. Drake, "to *popularize*" the Association with the profession in the United States, by transacting the business on purely republican principles.

As the Constitution stands, the ratio of representation is more than three times as great from Medical Schools, Hospitals, &c., as it is from the mass of the profession in County Medical Societies: this is productive of jealousy, and we hear the epithets of laymen and professors as at Cincinnati; or a jealousy exists between rival schools, and we have the professors of one school protesting against the admission of delegates from another Medical School, as was the case during the last meeting in Charleston.

Physicians are the best judges of the moral worth and professional respectability of their fellow practitioners; but by the present Constitution, the members of a County Medical Society may unanimously exclude a number of physicians from membership with them, and the rejected applicants may immediately organize themselves into a medical association, or obtain a charter for a medical school, and send one-third of their number as delegates to the American Medical Association.

Members of County Medical Societies now will not vote for Professors as delegates, because they consider they may be sent by their respective schools; and Professors neglect or refuse to join County Medical Societies, because they conceive they are excluded from its honors. But if the proposed alteration in the constitution is effected, professors who love their profession and feel an interest in the Association will join County Medical Societies, and, in return, the societies will be proud to send them as delegates, for they generally are, and always should be, the most intelligent and respectable of the profession.

Let the physicians of every county in the United States form themselves into a society, and have their constitution approved by the Censors of the State Society; let the constitutions of all State Societies be approved by Censors appointed by the American Medical Association; let every member of a county society be considered a member of the State Society and of the American Medical Association, and receive certificates of membership, and contribute to their funds. Then will we have realized the exalted ideas of Professor Meigs: we will have from ten thousand to forty thousand members, without any privileged classes among us, but each one will be entitled to his vote in the choice of delegates, and each one will contribute his share to the common fund; giving us an income of from ten thousand to two hundred thousand dollars, sufficient to reward labor and encourage enterprise, and thus give us incalculable influence and power.

A certificate of membership from the great "American Medical Association," will then be of more value to a young man than all the diplomas of all the medical schools in the country, for the inquiry will not be, is he a graduate? but, is he a member of the *American Medical Association?* Do the respectable physicians who are his immediate neighbors, and who are best qualified to judge, regard him as an intelligent, educated, honorable man, with whom they are willing to have professional and social intercourse? or do they regard him as an ignorant, uneducated man, or one who would resort to any means, however dishonorable, for the purpose of obtaining practice and popularity?

There are other arguments in support of the resolution, but it has been referred to a committee, the intelligent chairman of which, I have no doubt, will place the subject in its true light. I have only attempted to correct an error and to "define our position;" but as it is an important alteration in the constitution to be acted on at our next meeting, I think it is proper that the profession should discuss the matter, and that medical societies particularly should express their opinion.

Very respectfully, &c.,

*June* 15*th*, 1851. THOS. H. YARDLEY.

